# Association between Environmental Tobacco Smoke Exposure and Adaptive Behavior in Individuals with Autism Spectrum Disorder

**DOI:** 10.3390/toxics10040189

**Published:** 2022-04-13

**Authors:** Zofia Janik Szapuova, Lubica Argalasova, Diana Vondrova, Katarina Jansakova, Ivan Belica, Maria Kopcikova, Katarina Babinska, Daniela Ostatnikova

**Affiliations:** 1 Institute of Physiology, Faculty of Medicine, Comenius University, 813 72 Bratislava, Slovakia; zofia.szapuova@fmed.uniba.sk (Z.J.S.); katarina.jansakova@fmed.uniba.sk (K.J.); ivan.belica@fmed.uniba.sk (I.B.); maria.kopcikova@fmed.uniba.sk (M.K.); katarina.babinska@fmed.uniba.sk (K.B.); daniela.ostatnikova@fmed.uniba.sk (D.O.); 2 Institute of Hygiene, Faculty of Medicine, Comenius University, 813 72 Bratislava, Slovakia; diana.vondrova@fmed.uniba.sk; 3 Research Institute for Child Psychology and Pathopsychology, 831 05 Bratislava, Slovakia

**Keywords:** autism spectrum disorder, adaptive behavior, risk factors, environmental tobacco smoke, cotinine

## Abstract

The study focuses on current issues of adaptive behavior in individuals with autism spectrum disorder (ASD) and on the possible risk factor of environmental tobacco smoke (ETS). Children examined at the Academic Research Center for Autism (ARCA) in Bratislava were involved in the study. The study sample included 84 children (71 boys) with ASD (average age 5.35 years) and a non-ASD group of 24 children (20 boys; average age 8.10 years). The “ETS Questionnaire” focused on the detection of parental smoking habits and other ETS exposures. The concentrations of cotinine in urine were measured by ELISA kit. A significant delay in adaptive behavior of children with ASD in comparison with the non-ASD group was identified. The significant differences were in adaptive behavior, communication, and everyday skills. Children with ASD were more likely to be exposed to ETS, especially in the household. Good agreement was found between objective and subjective ETS exposure indicators (kappa = 0.613). Self-reported exposure to ETS corresponded significantly with the median levels of urinary cotinine. In addition to evaluation and assessment of the quality of adaptive behavior, an important goal of further research should be to identify, investigate, and eliminate environmental factors that interfere with adaptive behavior.

## 1. Introduction

Autism spectrum disorder (ASD) is considered to have multifactorial etiology; genetic factors in interaction with environmental factors are supposed to be the major players. The ability to adaptively behave relates to behavior and action in accord with common expectations. Adaptive behavior reflects the ability to perform everyday tasks necessary to move towards independent living. Research on adaptive behavior in autism is increasingly becoming a subject of interest in clinical practice, including intervention and therapeutic strategies in individuals with autism. Research on adaptive behavior in people with ASD focuses on identifying strengths and weaknesses within different areas of adaptive behavior, such as communication, everyday skills, and socialization, and on comparing these skills with the age-equivalent neurotypical population as well as individuals with other neurodevelopmental disorders. Many studies have found the greatest delays in socialization, followed by communication, while everyday skills appear to be the least affected area [1,2,3,4]. Individuals with autism display a significant delay in overall adaptive behavior as well as in all the aforementioned areas, according to standardized norms in a typically developed population. Adaptive functioning or behavior could be affected by various factors, such as cognitive abilities, language and motor skills, core symptoms of ASD, or co-occurring comorbidities. To improve the planning and implementation of the intervention, it is necessary to identify these factors. The importance of monitoring and evaluating the quality of adaptive behavior in autism is also supported by the changing of the classification of ASD in the EU according to the latest edition of the International Classification of Diseases (ICD-11), which was implemented on 1 January 2022 [5]. Adaptive behavior in people with ASD in Slovakia has not been sufficiently researched so far.

One of the significant environmental risk factors is environmental tobacco smoke (ETS) exposure. There has been increasing research on the effects of ETS on the health condition of children using subjective and objective study methods [6,7,8]. Nicotine’s major metabolite, cotinine, is the most appropriate objective marker for measuring passive cigarette smoke exposure [9,10,11]. Simultaneously, it is the most extensively utilized biomarker for identifying active and passive smokers [12]. In the liver, about 80–85% of nicotine is metabolized and transformed to cotinine [10]. Cotinine has a half-life of 37 to 160 h in children’s bodies, while nicotine has a half-life of 30 to 110 min. Cotinine is thus seen as an indicator of long-term tobacco exposure, whereas nicotine is thought to be an indicator of recent tobacco exposure [12,13]. Cotinine is present in the plasma, saliva, and urine of exposed individuals. In humans, it is excreted into the urine, mainly as *trans*-3′-hydroxycotinin [10]. Total tobacco smoke exposure is estimated using the resulting measured values [14]. Exposure to ETS includes both direct tobacco smoke and residual tobacco smoke found on smokers’ clothing and hair and on household surfaces [15]. A direct relationship between smoking during pregnancy and ASD has not been established. A recent meta-analysis [16] did not show a significant association between mothers’ smoking and the etiopathogenesis of autism in their children, nor did previous studies [17,18,19,20,21].

In recent years, there has been growing interest in research into the effects of passive smoking on the mental health of individuals. The results of several studies suggested an association between secondhand smoke and mental disorders [7,22,23,24] and indicated that exposure to tobacco smoke, whether prenatal or postnatal, increases the risk of problem behavior as well as the risk of cognitive impairment in children [6,25]. A meta-analysis performed by Chen et al. [26] showed that postnatal exposure to ETS was associated with poorer school performance and poorer cognitive performance in older children and adolescents. The authors also revealed an association between passive smoking and neurodevelopmental delay. Higher levels of cotinine were linked to symptoms of depression, anxiety disorder, and ADHD as well as problem behavior in children and adolescents, in a study by Bandiera et al. [7]. In this large population-based study involving 17,571 participants, children who were exposed to passive smoking at any level were at increased risk of mental health issues such as emotional problems, problem behaviors, hyperactivity and inattention, and problematic peer relationships [27]. ETS was found to be linked to cognitive impairment, negative mood, aggressive behavior, and insomnia in another study [28]. Salem et al. [29] revealed links between ETS exposure (as measured by the questionnaire method and cotinine measured in urine) and attention and visual–motor control.

The studies mentioned above were performed on the general population. There has been relatively little research on the possible relationship between ETS exposure and behavioral manifestations in the ASD population. One of the possible pathophysiological interpretations of this relationship is the effect of nicotine on nicotinic acetylcholine receptors. Nicotine exposure induces structural and synaptic changes in the developing brain, and these changes may persist into later life [30]. Another possible mechanism of action of nicotine is a change in the dopaminergic system; nicotine is known to stimulate phasic dopamine release in the striatum. Dopamine is an important neurotransmitter that is involved in controlling several aspects of emotional, behavioral, and cognitive functions [31].

Kim et al. [32] examined the link between cotinine levels and the manifestations of ADHD and ASD symptoms. According to their findings, higher urine cotinine levels were related to weaker adaptability and cognitive function outcomes. Based on their findings, the authors concluded that passive smoking had a deleterious impact on the manifestation of both ASD and ADHD symptoms [32]. In a recent cross-sectional study, Yang et al. [33] investigated the association between early-life exposure to ETS and autistic-like behaviors in a large group of preschoolers. Questionnaires completed by the mothers were used to assess ETS exposure. The findings demonstrated that preschoolers who were exposed to ETS early in life were more likely to exhibit autistic-like behaviors than preschoolers who were not exposed to ETS at any point in their early lives. Furthermore, the probability of autistic-like behaviors increased as the period of exposure and the average number of cigarettes smoked in the child’s environment increased [33].

The current study focused on adaptive behavior in individuals with ASD, with a special focus on the identification of risk factors, especially biological, that may be related to adaptive behavior. The important risk factor that attracted extensive attention was ETS exposure in the family and the child’s household.

The study hypotheses were as follows:

1. We assumed that children with ASD showed a significant lag in adaptive behavior compared with children suffering from nonspecified developmental difficulties and the neurotypical population.
2. We hypothesized a significantly negative relationship between ETS exposure and adaptive behavior in children with ASD.

The important research questions were as follows:

1. What is the profile of adaptive behavior in children with ASD in Slovakia compared with the standardized norms?
2. What is the agreement between objective and subjective indicators of exposure to environmental tobacco smoke?

## 2. Materials and Methods

### 2.1. Sample

Children diagnosed in the Academic Research Center for Autism (ARCA) at the Institute of Physiology, Faculty of Medicine, Comenius University Bratislava in Slovakia were involved in the study.

**Figure 1:**
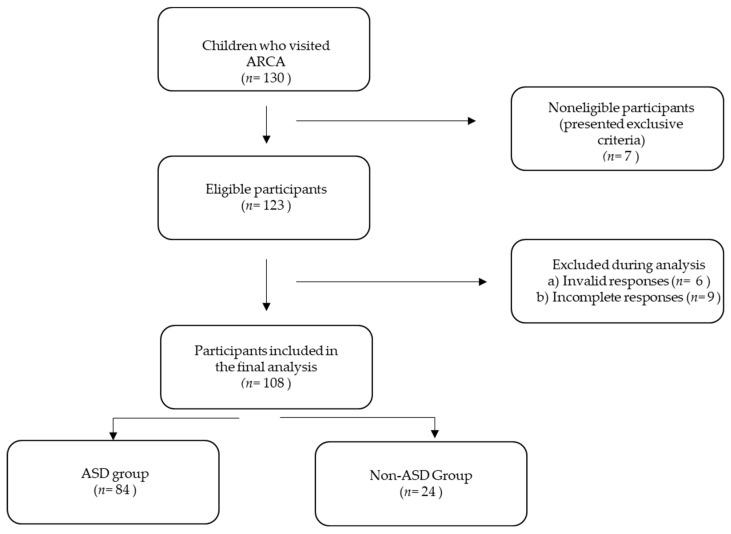
Flowchart diagram of subjects’ recruitment. ARCA—Academic Research Center for Autism; ASD—autism spectrum disorder.

A total of 130 children visited ACVA during one year. The inclusion criteria for the study sample for the assessment of ETS exposure in individuals with ASD was to meet the ICD-10 criteria for confirmation of ASD. The diagnosis was confirmed by two trained diagnosticians. Seven possible participants were excluded. The exclusion criteria were the presence of severe psychiatric and/or neurological comorbidities and the presence of other diseases with a genetic cause. Fifteen respondents were excluded because of invalid and incomplete responses. The final sample for the analysis consisted of 108 participants, 84 children (71 boys and 13 girls) with ASD (average age 5.35 years) and a group of 24 children (20 boys and 4 girls) without ASD (non-ASD group) (average age 8.10 years) (Figure 1 and Table 1). The sample size was based on the 80% power of the study.

Children in the non-ASD group were not typically developing. Parents of these children visited our center because of their concern about their development. In some cases, they were referred by other healthcare providers or specialists. Children in the non-ASD group had social and/or language difficulties. Diagnosis of ASD in this group was ruled out by the standard diagnostic tools used in our center (ADOS-2, Autism Diagnostic Observation Schedule—Second Edition; ADI-R, Autism Diagnostic Interview—Revised).

### 2.2. Diagnostic Evaluation of ASD

The Autism Diagnostic Observation Schedule—Second Edition (ADOS-2) [34] is a standardized assessment tool that helps providers diagnose ASD in children and adults. The ADOS-2 involves a semistructured play or interview session determined by the age and communication level of the individual. Deficits in four areas are evaluated: language and communication skills, social interaction, play, and creativity, the latter in terms of stereotyped, restricted, and repetitive behavior. Suitable for children and adults, the ADOS-2 has five modules depending on the individual’s age and language, and developmental level. In our study, participants were evaluated by module one, two, or three. The final score was divided into two domains: social affect (SA) and restricted and repetitive behavior (RRB). The sum of the scores in these domains gave an overall gross score, which allowed us to determine ASD diagnosis. The calibrated severity score (CSS) was created to better describe autism symptom severity consistently across different ages and language levels. The CSS has been widely used to quantify and compare symptom severity on a 10-point scale across modules.

The Autism Diagnostic Interview—Revised (ADI-R) [35] is a semistructured interview with the child’s parent (or primary caretaker) used for diagnosing autism, planning treatment, and distinguishing autism from other developmental disorders. It is currently one of the most proven methods, and together with ADOS-2, it is considered the “gold standard” in the diagnosis of ASD. ADI-R, a diagnostic interview with a parent, consists of three scales. Scale A (ADIR_A) represents qualitative abnormalities in reciprocal social interaction, scale B (ADIR_B) captures qualitative abnormalities in communication, and scale C (ADIR_C) identifies repetitive and stereotypical behaviors. Each item is scored based on the response of the parent/caretaker. Based on the determined severity level, the administrator assigns a value of 0 to 3 to each question. Subsequently, using the diagnostic algorithm, the resulting score is calculated. If these values exceed the cutoff scores obtained by standardization, the presence of an autism spectrum disorder is likely. Herein, scores were normalized to the min–max interval of 0 to 1 because of the different numbers of items counted. The final score represented the ratio of the obtained gross score and the maximum possible value that the child could obtain from all counted and evaluated items.

### 2.3. Adaptive Behavior Assessment: Vineland Adaptive Behavior Scale (VABS-3)

For assessing adaptive behavior, we used VABS-3 [36]. VABS-3 is a questionnaire consisting of three main scales, Communication (COM), Daily Living Skills (DLS), and Socialization (SOC), that correspond to the three broad areas of adaptive functioning specified by the American Association for Mental and Developmental Disabilities and the DSM-5 [36]. VABS-3 also includes a range of motor skills and maladaptive behavior, which are optional to fill in and do not count in the overall Adaptive Behavior Composite score (ABC). The Vineland scales have a strong historical background and are suitable for educational, clinical, and research purposes. They are standardized on a large and representative sample and provide an extensive view of the areas of adaptive functioning [37].

The VABS-3 is designed to be administered to respondents who are knowledgeable about the examinee’s everyday behavior. The responses are scored based on a Likert-type format with scores 0 (*never*), 1 (*sometimes*), and 2 (*usually or often*) that reflect the frequency to which an examinee performs an indicated behavior without help or prompting when that behavior is needed. Some items also require that a respondent answer *yes* (scored as 2) or *no* (scored as 0). The examinee needs to perform the activity independently.

The normative mean (for overall adaptive scores, communication, daily skills, socialization, and motor skills) adaptive behavior composite score is 100, with a standard deviation (SD) of 15. For the normative mean of each of the nine subscales that are included in the main scale scores, a value of 15 with a standard deviation of 3 was set. The scales of maladaptive behavior also had a normative mean of 15 with SD 3.

### 2.4. Assessment of ETS Exposure

For the subjective assessment of ETS exposure in our sample, the Parent Smoking Habits Questionnaire (ETS Questionnaire) was developed according to the questionnaires used in the studies of Sobotova et al. [38], utilizing nationally representative data from the 2000–2004 Medical Expenditure Panel Survey, and Sevcikova et al. [6], focusing on the detection of parental smoking habits, the prevalence and intensity of household smoking, and the other possible ETS exposures (mother: smoking before and during pregnancy, exposure to ETS during pregnancy, current smoking, smoking habits; father: smoking during wife’s pregnancy, smoking today, smoking habits; household: smoking in the household, number of cigarettes smoked per day in the household, place of smoking in the household; child: frequency and duration of exposure to ETS). One of the questions was, ‘‘Do you smoke?’’, and based on the answers submitted, parents were categorized as smokers, nonsmokers, and passive smokers.

The Questionnaire was filled in by parents or caregivers. The questionnaire was pilot validated with 20 respondents before the questions were finalized. Given that parents’/caregivers’ statements about their smoking habits may not always be reliable [14], it was necessary to use objective measurement to determine the degree of exposure.

### 2.5. Urine Cotinine Concentration Level Measurement

For the objective assessment, the concentration of cotinine in urine was measured using an ELISA method, the Calbiotech Cotinine Direct ELISA kit. The procedure was performed according to the instructions of the manufacturer. Briefly, 10 µL of standards and samples were transferred into the microtiter plate. Thereafter, 100 µL of enzyme conjugate was added to all wells. The whole plate was briefly mixed and subsequently incubated for 60 min at room temperature. After incubation, the content of the plate was discarded, and the plate was 6 times washed using distilled water in a volume of 350 µL/well. Then, 100 µL of substrate reagent was added to all wells, and the covered plate was incubated again at room temperature for 30 min. After incubation, the reaction was stopped using 100 µL of Stop reagent. The absorbance was measured at wavelengths of 450 nm and 650 nm.

### 2.6. Statistical Analysis

Descriptive statistics were used to describe the research sample and its clinical characteristics. We described the data using the central values, minimum, maximum, mean, standard deviation (SD), median, and interquartile range. The association between variables was assessed using Spearman and Pearson correlation coefficients. When comparing the values between the groups of quantitative data, we used the Student’s *t*-test/Mann–Whitney U test; for categorical variables, we used the chi-square test/Fisher’s test. The results were considered statistically significant at α levels < 0.05.

Descriptive and analytical statistics (categorical data analysis) were employed to identify associations between factors assessed in the questionnaire and self-reported ETS exposure. Kappa statistics, sensitivity, specificity, positive predictive value (PPV), and negative predictive value (NPV) were used to determine the accuracy of self-reported ETS exposure concerning the levels of urinary cotinine.

The kappa coefficient represented the percentage agreement between the parents’ subjective assessment of ETS exposure of their children and the accurate laboratory determination of their level of cotinine in the urine, considering the chance agreement [39].

There were missing data in some variable categories. There was no significant difference in terms of occurrence of missing data between ASD and non-ASD children. We considered these data as random missing and not affecting the reported associations. Missing data were not included in the analysis.

### 2.7. Ethical Approval

The study protocol was approved by the Ethics Committee of the Faculty of Medicine, Comenius University in Bratislava, Slovakia (33/2021). Participation in the research was voluntary and free of charge. The legal representatives of the participants were informed in advance about the necessary details concerning the research, and the children were included in the research only after informed consents were signed by their legal representatives.

## 3. Results

Clinical core symptomatology of ASD was characterized by the ADI-R and ADOS-2 diagnostic scales. Based on the overall calibrated score, children with ASD showed moderate to high severity of symptoms. The severity of clinical symptoms evaluated by ADI-R and ADOS-2 is shown in Table 2.

Adaptive behavior was assessed by the Vineland Adaptive Behavior Scale (VABS-3) [36]. Descriptive statistics for the ASD and non-ASD groups in terms of the total ABC score, the three main scales (Communication, Daily Living Skills, and Socialization), and motor skills are shown in Table 3.

The ASD group, on average, achieved a score below the normative range in all monitored areas. The adaptive behavior composite score averaged 69.6, which was up to two SDs below the norm. The children reached the best scores in motor skills (average = 81.75), followed by everyday skills (average = 75.4), socialization (average = 66.39), and communication (average = 65.76). Participants with ASD scored behind the standard of the neurotypical population by up to two standard deviations in overall adaptive behavior, socialization, and communication. Girls scored slightly better than boys. As with the main scales, children with ASD lagged significantly behind their healthy peers. We found the following average scores (ranking by severity): gross motor skills (13.09), domestic skills (11.41), fine motor skills (11.23), personal skills (10.58), written communication (10.41), community skills (9.85), coping (9.31), play and leisure time (8.95), expressive communication (8.44), interpersonal relationships (8.27), and receptive communication (8.04) (Table 3).

ETS exposure in the ASD and non-ASD samples was evaluated subjectively by a questionnaire based on parental reports. Children with ASD were more likely to be exposed to tobacco smoke according to their parents (*p* = 0.053). In the ASD group, 57% of children were exposed to ETS. Children with ASD were more likely exposed to tobacco smoke as subjectively reported by their parents; those children were also exposed to ETS in the household (*p* < 0.05). There were no significant differences for other indicators of subjective ETS exposure concerning the presence of ASD between the two samples of children (Table 4).

An important research question in our work was to determine the degree of agreement between subjective assessment of children’s ETS exposure based on parental statements and objective assessment by accurate determination of the concentration of cotinine concertation in the urine of participants by kappa statistics [39]. Good agreement was found between objective and subjective indicators of exposure to ETS (kappa = 0.613; specificity = 89.29; predictive value positive = 87.05; diagnostic accuracy = 82.22) (Table 5). Self-reported exposure to ETS also corresponded significantly with the median concentration of urinary cotinine (*p* < 0.001) (Table 6).

Table 6 shows the occurrence of subjective ETS exposure according to the level of urinary cotinine concentration. We compared the sample with a cotinine concentration below the median and the sample with a cotinine concentration above the median. We found a statistically significant difference in all monitored parameters except for the mother’s smoking before her pregnancy.

The median value of cotinine and interquartile range in children with ASD was 1.08 ng/mL (0.41; 2.64), and in children without ASD, 0.67 ng/mL (0.41; 2.63). We did not find any significant correlations between urinary cotinine concentration and the adaptive behavior functioning measured by VABS-3 in the ASD and non-ASD groups. The diagnostic tools ADI-R and ADOS-2 were used only to confirm/rule out the diagnosis of ASD in our study sample. We did not find significant correlations between urinary cotinine concentrations in ASD and non-ASD groups even after controlling for age and sex. All the respondents were Caucasian, and there were no other ethnic groups in our sample.

## 4. Discussion

To our best knowledge, this might be one of the first studies investigating the association between ETS exposure and adaptive behavior in children with ASD. Our study also focused on the profile of adaptive behavior in children with ASD in Slovakia compared with that in children without ASD and the standardized norms. An important research question was the degree of the agreement between objective and subjective indicators of exposure to ETS.

We found significantly weaker adaptive skills in ASD children than in their peers with typical neurodevelopment, which was consistent with previous research [1,3,4,40,41]. Based on our results, the weakest area of the three main areas of adaptive behavior in ASD children was communication, followed by socialization and everyday skills. Motor skills appeared to be the least affected area. These results also supported the findings of several authors [40,42,43] who observed a similar profile of adaptive behavior in children with ASD in their studies. We found the weakest subscale scores in interpersonal relationships, consistently with several studies that analyzed VABS-3 subscales [1,2,44].

Several studies have compared children with ASD and children with developmental delay without ASD [4,40,41]. Mouga et al. [41] compared the adaptive abilities of children with ASD and children with other neurodevelopmental disorders, and they found that the ASD group was characterized by significantly lower scores in the scales of daily living skills and socialization, though they did not find any significant difference in communication between groups. Nevill et al. [40] in their research observed a group of children with ASD (*n* = 122) and a non-ASD group (*n* = 36). The children in the non-ASD cohort were defined as those with speech disorders and with global developmental delays. That research sample was very similar to ours in this study.

In our study, we compared the adaptive behavior of ASD children with that of non-ASD children in all VABS-3 scales. We observed the most significant differences in the subscales of receptive and expressive communication and personal skills. Significant differences between the two groups were found in overall adaptive behavior, communication, daily living skills, and motor skills, as well as community skills and fine motor skills. Other differences were observed in the subscales of play and leisure time and gross motor skills. These results were partly consistent with the results of studies mentioned above [40,41].

An important research objective in our study was to examine the relationship between ETS exposure and adaptive behavior in children with ASD. At present, there is already relatively robust scientific evidence on the independent relationship between ASD and prenatal exposure to tobacco smoke [16,18,19,45]. Despite several studies on early exposure to ETS and its neurodevelopmental effects in the healthy pediatric population [30,46,47,48,49], relatively few studies have addressed this relationship in populations with ASD, and the findings of several existing studies were ambiguous [32,50,51,52].

In our research, we focused on the extent of ETS exposure in children with ASD compared with non-ASD children and on the relationship between ETS and behavioral parameters in the ASD sample. We assessed children’s exposure to ETS using both objective and subjective methods. An important research goal was to determine the degree of agreement between subjective assessment of children’s ETS exposure based on parental statements and objective assessment by accurate determination of the concentration of cotinine in the urine. To answer this question, we calculated the kappa coefficient. The kappa coefficient, in our case, exceeded the limit for good agreement, which meant significant agreement between the two methods. We consider such an approach to be important given that many researchers have chosen only one method and that there have been relatively few studies that combined them [8,21,29,53,54].

We did not find any significant correlations between urinary cotinine concentration and the adaptive behavior functioning measured by VABS-3 in the ASD and non-ASD groups. The diagnostic tools ADI-R and ADOS-2 were used only to confirm/rule out the diagnosis of ASD in our study sample. We did not find significant correlations between urinary cotinine concentrations in ASD and non-ASD groups even after controlling for age and sex. All the respondents were Caucasian, and there were no other ethnic groups in our sample.

However, according to self-report, autistic children were exposed to tobacco smoke more often than non-ASD children. Self-reported exposure to ETS corresponded significantly with median levels of urinary cotinine. Good agreement was found between objective and subjective ETS exposure indicators (kappa = 0.613).

In the scientific literature, we did not encounter research that specifically addressed this relationship. Research on ETS and ASD risk has been inconsistent in its conclusions. Khalil et al. [50] observed an increased risk of ASD in a sample of 56,710 children postnatally exposed to ETS, but only in boys. In contrast, Kaur et al. [17] (2014) did not observe any significant relationship between ETS exposure and ASD in a similarly large sample. To date, the only study to examine the relationship between behavioral parameters and passive smoking was the study by Kim et al. [32]. The results of this study suggested that cotinine levels predicted ASD compared with the control group, with an odds ratio of 1.89. The authors of this study reported that higher urinary cotinine levels were associated with problem behavior and impaired attention. However, they assessed behavior using different assessment tools (BASC-2, Behavioral Assessment System for Children, second edition). They checked also for ADHD with the Korean version of the ADHD rating scale. The association between urinary cotinine levels and an odds ratio of 1.55 for ADHD indicated an association between ETS and ADHD symptoms as well.

### Strengths and Limitations

The first limitation of our study was the lack of data on the intellectual abilities of the children and age differences between the study groups. Another limitation was the fact that we did not detect exact information about children’s developmental difficulties in the non-ASD group. As the non-ASD children visited our center for the ASD diagnostic process, a certain degree of linguistic, social, and educational difficulties could be assumed from parental reports. Furthermore, although cotinine is a reliable biomarker of exposure to ETS, other harmful substances in tobacco smoke need to be considered. The next limitation was the lack of information about the educational and socioeconomic status of the interviewed families, which may also be related to ETS exposure in the household. A further limitation of the study was that the age difference between the ASD group and the control group was significant. The children who did not have ASD were older. However, we did not find significant correlations between urinary cotinine concentrations in the ASD and non-ASD groups even after controlling for age. We found no significant differences between the two groups in terms of gender, low birth weight, or birth before the 37th week of pregnancy. All the respondents were Caucasian, and there were no other ethnic groups in our sample. The strength of the study is that this was one of the first studies investigating the association between ETS exposure and adaptive behavior in children with ASD. ETS exposure was assessed subjectively and objectively, and good agreement was found between objective and subjective ETS exposure indicators.

In further research, we plan to expand our sample and add the control group of typically developing children. Evaluation and assessment of the quality of adaptive behavior should become an integral part not only of the autism diagnostic process but of planning effective interventions. Various behavioral and psychosocial interventions are known to improve certain aspects of behavioral manifestations and social interactions and to develop language skills in ASD individuals. There is no doubt about the importance of early diagnosis of this disorder, but it is equally important to assess the quality of adaptive abilities, which should be monitored longitudinally, as the quality of these abilities helps to predict the prognosis of an individual with autism.

## 5. Conclusions

In our study, we identified a significant delay in adaptive behavior of children with ASD in comparison with the non-ASD group. Significant differences were revealed in overall adaptive behavior, communication, and everyday skills. These findings are important from the differential diagnosis point of view. Subjective exposure to ETS and smoking in the household were significantly related to the presence of ASD. Good agreement was found between objective and subjective indicators of exposure to ETS in our study. In addition to evaluation and assessment of the quality of adaptive behavior, an important goal of further research should be to identify, investigate, and eliminate environmental factors that interfere with adaptive behavior.

## Figures and Tables

**Table 1:** Demographic characteristics of the sample.

Characteristic		ASD Group (*n* = 84)	Non-ASD Group (*n* = 24)	*p*-Value ^^^
**Age, mean (SD)**		5.4 (3.0)	8.1 (3.6)	<0.001 ***
**Sex, N (%)**	female	13 (15.5)	4 (16.7)	n.s.
	male	71 (84.5)	20 (83.3)	n.s.
**Order of birth, N (%)**	1	40 (47.6)	13 (54.2)	<0.05 *
	2	33 (39.3)	7 (29.2)	n.s.
	3	6 (7.1)	3 (12.5)	n.s.
	4	5 (6.0)	1 (4.2)	n.s.
**Preterm birth, N (%)**	no	77 (91.7)	22(91.7)	n.s.
	yes	7(8.3)	2(8.3)	n.s.
**LBW, N (%)**	no	80 (95.2)	22 (91.7)	n.s.
	yes	4 (4.8)	2 (8.3)	n.s.

^^^*t*-test, Fisher’s exact test; n.s.—nonsignificant; *, ***—statistically significant, very highly; ASD—autism spectrum disorder; SD—standard deviation; LBW—low birth weight.

**Table 2:** The severity of clinical symptoms of ASD group (*n*= 84).

Diagnostic Tool	Total N	Minimum	Maximum	Mean	Standard Deviation
**ADIR_A**	84	0.1	0.9	0.5	0.2
**ADIR_B**	84	0.1	0.1	0.5	0.2
**ADIR_C**	84	0.0	0.8	0.3	0.2
**ADOS_SA**	84	3.0	10.0	7.3	1.6
**ADOS_RRB**	84	2.0	10.0	8.6	1.4
**ADOS_CSS**	84	4.0	10.0	8.0	1.6

ASD—autism spectrum disorder; ADIR_A—qualitative abnormalities in reciprocal social interaction; ADIR_B—qualitative abnormalities in communication; ADIR_C—restricted, repetitive patterns of behavior and interests; ADOS_SA—social affect; ADOS_RRB—restricted and repetitive behavior; ADOS_CSS—calibrated severity score.

**Table 3:** Adaptive behavior profile (main domains and subdomains) in ASD and non-ASD participating children.

Main Domains	Non-ASD Group Mean (SD)	ASD Group Mean (SD)	*p*-Value ^^^
**Adaptive Behavior Composite**	78.6 (15.6)	69.6 (10.8)	<0.05 *
**Communication**	80.9 (16.6)	65.8 (19.2)	<0.01 **
**Daily Living Skills**	85.1 (15.7)	75.4 (11.9)	<0.01 **
**Socialization**	72.4 (18.4)	66.4 (14.6)	n.s.
**Motor skills**	90.2 (14.5)	81.8 (10.8)	<0.01 **
**Subdomains**	**Non-ASD Group** Mean (SD)	**ASD Group** Mean (SD)	***p*** **-Value**
**Receptive**	12.0 (3.4)	8.0 (4.4)	<0.001 ***
**Expressive**	12.2 (3.7)	8.4 (4.6)	<0.001 ***
**Written**	11.0 (3.3)	10.4 (3.3)	n.s.
**Personal**	12.9 (2.9)	10.6 (2.5)	<0.001 ***
**Domestic**	12.3 (3.2)	11.4 (2.2)	n.s.
**Community**	11.8 (3.3)	9.9 (2.9)	<0.05 *
**Interpersonal relationships**	9.2 (3.4)	8.3 (2.8)	n.s.
**Play and leisure time**	10.7 (3.9)	9.0 (3.4)	<0.05 *
**Coping skills**	9.8 (3.4)	9.3 (2.4)	n.s.
**Gross motor**	14.0 (2.9)	13.1(2.0)	<0.05 *
**Fine motor**	13.0 (2.8)	11.0 (2.9)	<0.01 **

^^^ *t*-test or Mann–Whitney U test; n.s.—nonsignificant; *, **, *** —statistically significant, highly, very highly; ASD—autism spectrum disorder; SD—standard deviation.

**Table 4:** Subjective ETS exposure relating to the presence of ASD (*n* = 54).

Indicators of Subjective ETS Exposure		ASD				*p*-Value ^^^
		No		Yes		
		N	%	N	%	
**Subjective ETS exposure of the child**	**No**	12	85.7	23	57.5	0.05 *
	**Yes**	2	14.3	17	42.5	
**Smoking of mother before pregnancy**	**No**	13	92.9	33	82.5	n.s.
	**Yes**	1	7.1	7	17.5	
**Smoking of father during mother’s pregnancy**	**No**	12	85.7	30	75.0	n.s.
	**Yes**	2	14.3	10	25.0	
**ETS exposure of mother during pregnancy**	**Not at all**	12	85.7	22	56.4	n.s.
	**Once a week**	2	14.3	10	25.6	
	**More times a week/every day**	0	0.0	7	17.9	
**Current smoking of the mother**	**No**	13	92.9	31	77.5	n.s.
	**Yes**	1	7.1	9	22.5	
**Current smoking of the father**	**No**	12	85.7	27	69.2	n.s.
	**Yes**	2	14.3	12	30.8	
**Smoking in the household**	**No**	13	92.9	25	62.5	<0.05 *
	**Yes**	1	7.1	15	37.5	
**Number of cigarettes smoked at home**	**Not at all**	13	92.9	24	60.0	n.s.
	**≤10**	1	7.1	10	25.0	
	**>10**	0	0.0	6	15.0	

^^^ Fisher’s exact test; n.s.—nonsignificant; *—statistically significant; ASD—autism spectrum disorder; ETS—environmental tobacco smoke.

**Table 5:** Agreement between subjective assessment of ASD and non-ASD group children’s ETS exposure based on parental statements and objective assessment by accurate determination of cotinine concentration in urine (*n* = 45).

Subjective vs. Objective ETS Exposure	
Measures of Agreement	Total
**Kappa**	0.613
**Spearman correlation**	0.62
**Sensitivity**	70.59
**Specificity**	89.29
**Positive predictive value**	87.05
**Negative predictive value**	74.85
**Diagnostic accuracy**	82.22

ETS—Environmental tobacco smoke.

**Table 6:** Subjective ETS exposure comparing to the median concentration of urinary cotinine (*n* = 45).

Indicators of Subjective ETS Exposure		Concentration of Urinary Cotinine				*p*-Value ^^^
		Below the Median		Above the Median		
		N	%	N	%	
**ETS exposure of the child**	**No**	25	89.3	5	29.4	<0.001 ***
	**Yes**	3	10.7	12	70.6	
**Quantification of ETS exposure of the child**	**Not at all**	18	64.3	5	29.4	<0.01 **
	**Once a week**	9	32.1	5	29.4	
	**More times a week/every day**	1	3.6	7	41.2	
**Smoking of mother before pregnancy**	**No**	26	92.9	13	76.5	n.s.
	**Yes**	2	7.1	4	23.5	
**Smoking of father during mother’s pregnancy**	**No**	25	89.3	10	58.8	<0.05 *
	**Yes**	3	10.7	7	41.2	
**ETS exposure of mother during pregnancy**	**Not at all**	22	78.6	7	43.8	<0.05 *
	**Once a week**	5	17.9	6	37.5	
	**More times a week/every day**	1	3.6	3	18.8	
**Current smoking of the mother**	**No**	27	96.4	10	58.8	<0.01 **
	**Yes**	1	3.6	7	41.2	
**Current smoking of the father**	**No**	25	89.3	8	50.0	<0.01**
	**Yes**	3	10.7	8	50.0	
**Number of cigarettes smoked at home**	**Not at all**	26	92.9	5	29.4	<0.001 ***
	**≤10**	1	3.6	8	47.1	
	**>10**	1	3.6	4	23.5	
**Smoking in the household**	**No**	26	92.9	6	35.3	<0.001 ***
	**Yes**	2	7.1	11	64.7	

^ Fisher’s exact test; n.s.—nonsignificant; *, **, ***—statistically significant, highly, very highly; ETS—environmental tobacco smoke.

## Data Availability

Not applicable.
